# A Study on the Effect of Umbilical Cord Milking Along With Delayed Cord Clamping on Hematological Parameters in Comparison to Delayed Cord Clamping Alone in Moderate-to-Late Preterm Newborns

**DOI:** 10.7759/cureus.69256

**Published:** 2024-09-12

**Authors:** Pooja Dahiya, Shweta, Anand Parashar, Tejaswee Lohakare

**Affiliations:** 1 Department of Paediatrics, Kalpana Chawla Government Medical College, Karnal, IND; 2 Department of Paediatrics, Amrita Institute of Medical Sciences and Research Centre, Faridabad, IND; 3 Child Health Nursing, Srimati Radhikabai Meghe Memorial College of Nursing, Datta Meghe Institute of Higher Education & Research, Wardha, IND

**Keywords:** delayed cord clamping, hemoglobin, neonatal anemia, preterm infants, serum ferritin, umbilical cord milking

## Abstract

Background

Anemia, particularly iron deficiency anemia (IDA), is a global public health issue with serious implications for infant cognitive and developmental outcomes. Preterm infants are especially vulnerable to IDA due to reduced placental blood transfer at birth. Delayed cord clamping (DCC) and umbilical cord milking (UCM) are interventions aimed at enhancing this blood transfer, thereby improving neonatal iron status. While DCC allows passive blood transfer by delaying cord clamping, UCM actively expedites the process. However, there remains a lack of consensus on the comparative benefits of these methods, particularly in preterm infants. This study aims to clarify the efficacy of UCM combined with DCC versus DCC alone in improving hematological outcomes in moderate-to-late preterm newborns.

Methodology

This comparative study was conducted at Maharaja Agrasen Medical College, Agroha, Hisar, Haryana, over a 12-month period. The study included 200 moderate-to-late preterm infants (32-36+6 weeks of gestation), divided into two groups: Group A (DCC alone) and Group B (DCC combined with UCM). The study aimed to compare the effects of these two interventions on hematological outcomes. Data were collected on baseline characteristics, birth weight, hemoglobin (Hb) levels at birth and at six weeks, serum ferritin levels at six weeks, and any complications. Statistical analyses included independent t-tests for continuous variables and chi-squared tests for categorical variables to assess the differences between the two groups.

Results

There were no significant differences in the baseline characteristics, birth weight, or clamping time between the two groups. Mean Hb levels at birth were 15.46 g/dL in the DCC group and 15.72 g/dL in the DCC+UCM group (p = 0.429). At six weeks, the mean Hb levels were 13.10 g/dL for the DCC and 13.24 g/dL for the DCC+UCM (p = 0.541). Serum ferritin levels at six weeks were 239.26 ng/mL for the DCC and 258.06 ng/mL for DCC+UCM (p = 0.146). Complications were similar between the groups, with no significant differences in the rates of intraventricular hemorrhage (IVH), jaundice, or polycythemia.

Conclusion

In this study, the combination of UCM with DCC did not show significant differences in hematological outcomes compared to DCC alone in moderate-to-late preterm infants. Both interventions demonstrated similar results for hemoglobin and ferritin levels, and there were no notable differences in adverse outcomes. Further research with larger sample sizes and longer follow-ups is necessary to better understand the potential benefits of UCM in preterm neonates.

## Introduction

Anemia, particularly iron deficiency anemia (IDA), poses a significant public health challenge worldwide, affecting both developing and developed countries. Iron deficiency in infants can have profound long-term consequences, affecting cognitive and developmental outcomes [1]. Iron deficiency in the early months of life often results from inadequate iron stores transferred from the placenta at birth. This phenomenon is particularly critical for preterm infants who are at a higher risk of IDA due to reduced placental blood transfer and limited iron reserves [2]. Approximately 80 mL of blood is transferred from the placenta to the neonate within the first minute after delivery, with up to 100 mL being transferred within three minutes [3]. This blood transfer supplies the neonate with essential iron, which is crucial for preventing anemia during infancy. Delayed cord clamping (DCC) and umbilical cord milking (UCM) are two interventions aimed at optimizing this blood transfer and improving neonatal iron status [4].

DCC involves postponing the clamping of the umbilical cord for 30 to 180 seconds after birth, allowing for a passive transfer of blood from the placenta to the neonate [5]. This method has been shown to increase neonatal hemoglobin and iron stores, reducing the incidence of IDA in infants [6]. Conversely, UCM involves actively squeezing the umbilical cord to expedite blood transfer from the placenta to the neonate, potentially improving the efficiency of this process [7]. Recent studies have evaluated the efficacy of DCC and UCM in various neonatal populations. Katheria et al. [8] found that UCM improved hemodynamic parameters and blood volume in preterm infants delivered via a cesarean section, although these benefits were not observed in vaginal deliveries. Similarly, UCM is known to be associated with higher rates of severe intraventricular hemorrhage (IVH) than DCC, although the overall mortality and severe IVH rates were similar between the two groups [9]. Some studies show the benefits of UCM on hematocrit levels, but no significant differences in anemia rates or adverse outcomes have been reported [10].

By contrast, a Cochrane review found that DCC was associated with higher hemoglobin levels and lower rates of IDA than early cord clamping [4]. The American College of Obstetricians and Gynecologists (ACOG) and the American Academy of Pediatrics (AAP) recommend DCC for at least 60 seconds unless an emergency situation dictates otherwise [11]. UCM, on the other hand, may be advantageous in situations requiring rapid placental transfusion. Despite these findings, there remains a lack of consensus regarding the comparative benefits of UCM and DCC in preterm infants. This study aimed to compare the effects of UCM combined with DCC versus DCC alone on hematological outcomes in moderate-to-late preterm newborns. By investigating these methods, the study aimed to clarify their efficacy and potential advantages in improving neonatal iron status and reducing the risk of anemia.

## Materials and methods

Study design and setting

This comparative study was conducted in the Departments of Pediatrics and Obstetrics and Gynecology at Maharaja Agrasen Medical College (MAMC) in Agroha, Hisar, Haryana, over a 12-month period from December 15, 2020, to December 20, 2021. The study was initiated following approval from the Scientific Research Committee of Maharaja Agrasen Medical College, Agroha, Hisar, on December 10, 2020, under approval number MAMC/SRC/20/DNB/SRC/23.

Sample Size Justification

The sample size of 200 participants was chosen to ensure adequate power for detecting clinically significant differences between the DCC and DCC combined with UCM (DCC+UCM) groups. A power calculation was performed using an estimated effect size based on previous studies and expected differences in key outcomes such as birth weight, hemoglobin levels, and serum ferritin levels [3,4,6]. The power analysis assumed a significance level of 0.05 and a power of 80% to detect meaningful differences between the groups. Given the constraints of the study period and available resources, the sample size was set to 100 participants per group. This number was deemed sufficient to achieve statistical reliability and validity for the comparisons, accounting for potential dropout and variability within the data. The use of non-probability sampling aimed to ensure representative distribution and reliable analysis of outcomes.

Selection Criteria

The study included moderate-to-late preterm infants born between 32 and 36 weeks and six days of gestation. Both male and female infants were considered, and deliveries could be either vaginal or via lower-segment cesarean section (LSCS). Inclusion required an APGAR score greater than 7 at one minute after birth. Exclusion criteria for infants included those born before 32 weeks or after 37 weeks of gestation, multiple births, isoimmunization, birth asphyxia, respiratory distress, congenital anomalies, or resuscitation needs. Mothers with preterm premature rupture of membranes (PROM), maternal fever, chorioamnionitis, foul-smelling liquor, urinary tract infections, or excessive vaginal examinations were also excluded.

Data sources and variables

Data were collected from detailed history, clinical examination, and maternal case sheets. Key variables included baseline characteristics, birth weight, hemoglobin (Hb) levels at birth and at six weeks, and serum ferritin levels at six weeks. Complications such as intraventricular hemorrhage, late-onset sepsis, necrotizing enterocolitis, polycythemia, and hyperbilirubinemia were monitored within the first six weeks of life.

Statistical analysis

Data were analyzed using IBM SPSS Statistics for Windows, Version 23.0 (released 2014, IBM Corp., Armonk, NY). Continuous variables were summarized as means and standard deviations, while nominal and categorical variables were reported as frequencies and percentages. The independent t-test was used to compare normally distributed continuous variables, and the chi-square test was used for categorical variables. Statistical significance was set at a p-value of 0.05.

## Results

Table 1 presents the baseline characteristics of the patients in the study, comparing the DCC group and DCC+UCM group. The baseline characteristics, including socioeconomic status, gender distribution, gestational age, mode of delivery, and maternal and baby blood groups, showed no statistically significant differences between the DCC and DCC+UCM groups. Both groups were well-matched in terms of these factors, ensuring comparability in further analysis.

**Table 1:** Baseline characteristics of the patients DCC: delayed cord clamping, UCM: umbilical cord milking, W: weeks, LSCS: lower-segment cesarean section, NVD: normal vaginal delivery p < 0.05 considered statistically significant; Chi-square test applied

Baseline characteristics		DCC		DCC+UCM		Total		
		Count	Column %	Count	Column %	Count	Column %	P Value
Socioeconomic status	Lower	33	33.0%	37	37.0%	70	35.0%	0.83
	Lower middle	59	59.0%	56	56.0%	115	57.5%	
	Upper middle	8	8.0%	7	7.0%	15	7.5%	
Gender of the baby	Female	42	42.0%	52	52.0%	94	47.0%	0.16
	Male	58	58.0%	48	48.0%	106	53.0%	
Gestational age	32-34W	25	25.0%	30	30.0%	55	27.5%	0.43
	34-37W	75	75.0%	70	70.0%	145	72.5%	
Mode of delivery	LSCS	48	48.0%	49	49.0%	97	48.5%	0.89
	NVD	52	52.0%	51	51.0%	103	51.5%	
Mother's blood group	A+	17	17.0%	20	20.0%	37	18.5%	0.92
	AB+	10	10.0%	11	11.0%	21	10.5%	
	B+	41	41.0%	37	37.0%	78	39.0%	
	O+	32	32.0%	32	32.0%	64	32.0%	
Baby's blood group	A-	1	1.0%	0	0.0%	1	0.5%	0.92
	A+	17	17.0%	18	18.0%	35	17.5%	
	AB+	13	13.0%	14	14.0%	27	13.5%	
	B-	2	2.0%	3	3.0%	5	2.5%	
	B+	34	34.0%	35	35.0%	69	34.5%	
	O+	33	33.0%	30	30.0%	63	31.5%	

Table 2 represents the birth weight (in kg) of the babies. The mean birth weight of babies in both groups was similar, with no statistically significant difference (p = 0.405). The average birth weight in the DCC group was 2.39 kg, while the DCC+UCM group had a mean birth weight of 2.34 kg.

**Table 2:** Birth weight of the babies N: number of participants, DCC: delayed cord clamping, UCM: umbilical cord milking Independent t-test applied; p = 0.405 (not significant)

Group	N	Mean (kg)	Std. Deviation (kg)	Minimum (kg)	Maximum (kg)	Range (kg)
DCC	100	2.385	0.37534	1.7	3.3	1.6
DCC+UCM	100	2.34	0.38638	1.65	3.75	2.1
Total	200	2.3625	0.38062	1.65	3.75	2.1

Table 3 shows the mean clamping time in both groups. Both groups had an identical mean clamping time of 60 seconds, with no variation or statistical difference between the DCC and DCC+UCM groups.

**Table 3:** Time of clamping (in seconds) DCC: delayed cord clamping, UCM: umbilical cord milking

Measurement (in seconds)	DCC	DCC+UCM	Total
N	100	100	200
Mean	60	60	60
Std. deviation	0	0	0
Minimum	60	60	60
Maximum	60	60	60
Range	0	0	0

Table 4 presents the hemoglobin level (in gm/dl). There was no statistically significant difference in hemoglobin levels between the DCC and DCC+UCM groups at birth (p = 0.429) or at six weeks (p = 0.541). Both groups demonstrated similar hemoglobin trends over time.

**Table 4:** Hemoglobin level (in gm/dl) DCC: delayed cord clamping, UCM: umbilical cord milking p-value = 0.426 at birth and 0.541 at six weeks (not significant); independent t-test applied

Measurement (in g/dL)	DCC (Mean)	DCC (Std. Deviation)	DCC+UCM (Mean)	DCC+UCM (Std. Deviation)	p-value
Hb at birth	15.46	2.34	15.72	2.26	0.429
Hb at six weeks	13.1	1.59	13.24	1.57	0.541

Table 5 represents the serum ferritin level, which shows that the mean ferritin level was 239.26 in the DCC group, whereas in the DCC+UCM, it was 258.06. On the statistical analysis, no significant differences were found between the groups.

**Table 5:** Serum ferritin level DCC: delayed cord clamping, UCM: umbilical cord milking p-value = 0.146 (not significant; independent t-test applied

Measurement (serum ferritin level, µg/L)	DCC (Mean)	DCC (Std. Deviation)	DCC+UCM (Mean)	DCC+UCM (Std. Deviation)	Total (Mean)	Total (Std. Deviation)
Serum ferritin level (µg/L)	239.26	90.52	258.06	91.62	248.66	91.33

Table 6 summarizes the complications observed in the study groups, including the DCC group and the DCC+UCM group. Complications such as intraventricular hemorrhage (IVH), jaundice, and polycythemia were infrequent and showed no statistically significant differences between the two groups (p = 0.681).

**Table 6:** Complications DCC: delayed cord clamping, UCM: umbilical cord milking, IVH: intraventricular hemorrhage Fischer’s exact test applied; p = 0.681 (not significant)

Complications	DCC		DCC+UCM		Total	
	Count	Column %	Count	Column %	Count	Column %
IVH	1	1.0%	0	0.0%	1	0.5%
Jaundice	4	4.0%	3	3.0%	7	3.5%
Polycythaemia	1	1.0%	2	2.0%	3	1.5%

## Discussion

Anemia is a significant public health issue globally, particularly affecting young children and pregnant women in developing countries [12]. Evidence indicates that anemia in infancy and childhood can have long-term cognitive effects. IDA in infancy arises from insufficient iron stores at delivery [13]. The transfer of blood from the placenta to the newborn involves roughly 80 mL of blood, which is transferred within the first minute after delivery, reaching up to 100 mL within three minutes [5]. This process provides neonates with 40-50 mg/kg of iron, which helps prevent IDA during the first year of life [14]. Two primary methods are used to transfer blood from the placenta to the neonate. DCC involves clamping the umbilical cord 30 to 180 seconds post-delivery, which is regarded as a passive method of transfer [5]. Conversely, UCM entails the active compression of the cord from the maternal end toward the newborn, which is a dynamic transfer technique [15].

The hemoglobin concentration at birth and iron reserves in the initial months are thought to influence developmental results [16]. Nevertheless, delaying cord clamping for 30-60 seconds may be too lengthy in some emergency situations, such as non-reassuring fetal status or maternal hemodynamic instability. UCM is considered a method to achieve quicker placental blood transfer to the neonate, within 10-15 seconds [4]. However, there is limited data comparing the effects of UCM, DCC, and DCC alone in term and preterm neonates [17]. One study found no significant difference between cut cord milking (C-UCM) and DCC in term neonates [18]. The purpose of this study was to compare the effects of UCM combined with DCC versus DCC alone on hematological parameters in moderate to late preterm newborns aged six weeks. This hospital-based, single-center, comparative study included 200 women who delivered and were divided into two groups: Group 1 (DCC), which received delayed cord clamping for 60 seconds only, and Group 2 (DCC+UCM), which received delayed cord clamping for 60 seconds followed by UCM.

Katheria et al. [8] found that UCM in preterm infants delivered by a cesarean section resulted in higher superior vena cava flow, right ventricular output, hemoglobin, blood pressure, and urine output than DCC, although no differences were noted in vaginal deliveries. Katheria et al. [19] reported no significant difference in death or severe IVH between UCM and DCC groups, but UCM was associated with a higher rate of severe IVH (8% vs. 3%). Panburana et al. [5] found no significant difference in hemoglobin levels or adverse outcomes between term neonates receiving UCM or DCC. Mangala et al. [20] noted that UCM resulted in higher venous hematocrit at 48 hours and six weeks than DCC. Sura et al. [21] observed similar neonatal hemoglobin, hematocrit, and anemia rates between the UCM and DCC groups, but UCM was associated with lower rates of neonatal polycythemia and jaundice.

Kumbhat et al. [22] found that among infants born at <29 weeks’ gestation, those exposed to UCM had higher odds of severe IVH than those with DCC, although overall mortality and severe IVH rates were similar between groups. Balasubramanian et al. [23] concluded that UCM significantly increased the risk of severe IVH compared to DCC, but it did not improve clinical outcomes or reduce the need for transfusions. The advantages of DCC on blood parameters in full-term infants include higher hemoglobin and hematocrit levels, as demonstrated by Singh et al. [24]. The Cochrane review found DCC associated with higher hemoglobin levels and lower incidence of IDA compared to early cord clamping (ECC). The American College of Obstetricians and Gynecologists (ACOG) and the American Academy of Pediatrics (AAP) recommend DCC for at least 60 seconds unless an emergency arises. UCM is beneficial in emergencies involving rapid placental transfusions. Upadhyay et al. [25] reported that UCM resulted in higher hemoglobin and iron storage than ECC did. However, comparisons between DCC and UCM in term neonates are limited, and Jaiswal et al. [26] found no significant differences in hematological outcomes between the two methods. UCM's intact UCM method allows for quicker placental-to-neonate transfusions, making it suitable for both neonatal resuscitation and unstable maternal conditions.

Limitations of the study

The study had several limitations. The relatively short follow-up period of six weeks did not allow for a comprehensive assessment of long-term hematologic status and potential outcomes. Future research should consider extending the follow-up to six to 12 months to provide a more thorough evaluation of both benefits and adverse effects. Furthermore, while the study focused on hemoglobin (Hb) and ferritin levels, incorporating additional hematological parameters such as hematocrit, reticulocyte count, and bilirubin levels could offer a more complete picture of the neonatal hematological profile. In addition, the small sample size for reporting complications like IVH, jaundice, and polycythemia may limit the ability to detect statistically significant differences. These factors suggest the need for larger studies to validate the findings and provide a more robust understanding of the interventions' impacts.

## Conclusions

The study assessed the effects of UCM in comparison to DCC and DCC alone on hematological outcomes and complications in moderate-to-late preterm infants. The findings showed that UCM did not provide significant benefits over DCC alone in terms of hematological markers such as hemoglobin and serum ferritin levels. Both interventions yielded comparable results with no significant differences in adverse outcomes. Birth weights were similar across all groups. The low incidence of complications, including a single case of IVH in the DCC group, does not indicate a clear advantage of UCM in preventing these conditions. These results suggest that while UCM may not offer additional benefits over DCC in this context, further research with larger sample sizes and longer follow-up is necessary to fully understand its potential advantages and implications for preterm neonates.
